# Incidental Unruptured Isolated Left Common Iliac Artery Aneurysm: A Rare Entity

**DOI:** 10.7759/cureus.11588

**Published:** 2020-11-20

**Authors:** Mohamed Selim, Mohamed A Nasr, Moath T Alkhouzaie, Dinah A AlNoaimi, Mohammed H Alam

**Affiliations:** 1 Vascular Surgery, Al Azhar Faculty of Medicine, Cairo, EGY; 2 Vascular Surgery, King Fahad University Hospital, Dammam, SAU

**Keywords:** iliac artery aneurysm, unruptured aneurysm, iliac artery, incidental aneurysm

## Abstract

Iliac artery aneurysms (IAA) are a rare entity. The etiology behind IAA is unclear; however, it is typically degenerative or atherosclerotic in origin. In patients presenting with sudden rupture requiring emergent surgery, mortality rates are high, signifying the need for prompt diagnosis and treatment. We report a case of an incidentally found unruptured isolated left common iliac artery aneurysm in an 80-year-old man. Management with aortofemoral angioplasty was successfully performed for this patient.

## Introduction

An aneurysm is a focal dilation in a localized segment of an artery. When its diameter exceeds 50% of the normal vessel diameter, it could affect the common, internal, or external iliac arteries, and it could occur bilaterally. Iliac artery aneurysms (IAA) are found to coexist with abdominal artery aneurysms (AAA) in around 40% of AAA patients. Contrarily, isolated iliac artery aneurysms are a rare entity, and the rate of them occurring is estimated to be between 0.4% and 1.9% of abdominal aneurysms [1-3].

The etiology behind IAA is unclear; however, it is typically degenerative or atherosclerotic in more than 90% of cases. Other less common etiologies are infection, trauma, and connective tissue diseases. IAA commonly occurs in elderly males with a concomitant history of smoking and hypertension. Owing to the deep location of IAA in the pelvic cavity, they remain asymptomatic as they progress and are detected incidentally because of the increasing usage of ultrasound and CT. However, when IAA are large enough to compress nearby structures, symptoms will arise in the form of urinary symptoms, constipation, paresthesia, sciatica, and, less commonly, thrombosis. Rupture of IAA carries a high mortality risk, demonstrating the significance of prompt diagnosis and treatment [1-3].

In patients presenting with sudden rupture requiring emergent surgery, a mortality rate as high as 33%-59% is reported while the mortality rate in patients with an elective repair is less than 5%. Owing to the rarity of this pathology, no clear guidelines regarding asymptomatic patients' management were developed. Management methods for IAA include either open surgery or endovascular repair. The choice of management usually depends on the stability of the patient, operative risk, and the characteristics of the aneurysm. It is recommended to consider elective repair in aneurysms larger than 3.5 cm; however, in aneurysms larger than 5 cm, it is mandatory to intervene to reduce mortality and the potentially life-threatening sequelae associated with rupture [1,2,4].

Here, we report a case of an incidental, unruptured, isolated left common IAA in an 80-year-old man treated successfully with aortofemoral angioplasty.

## Case presentation

**Figure 1:**
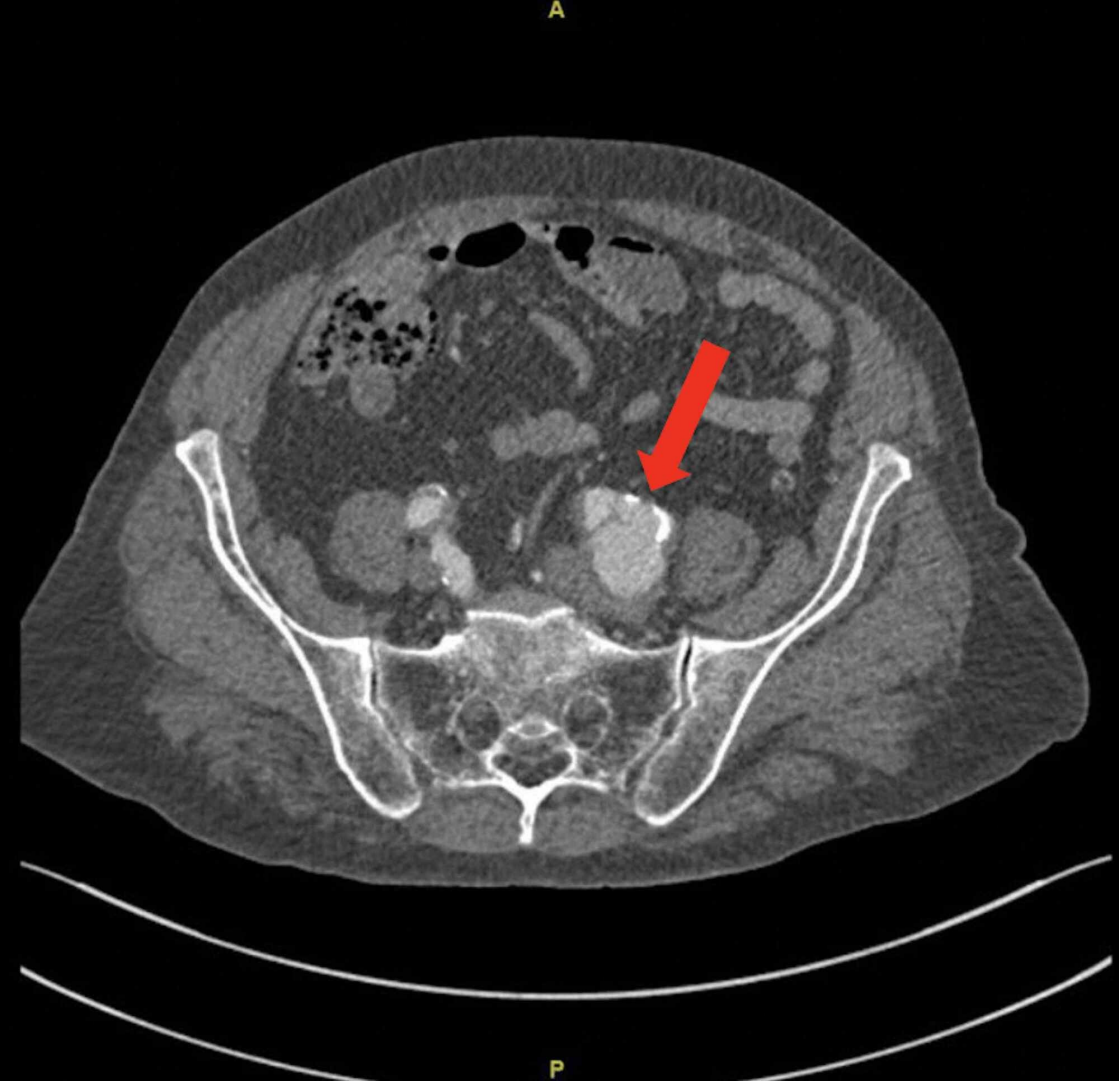
CT with contrast showing an isolated unruptured left common iliac artery aneurysm CT: computed tomography

An 80-year-old, married, Saudi male, known case of asthma, diabetes, hypertension, chronic kidney disease (CKD), and ischemic heart disease (IHD), with a history of percutaneous coronary intervention (PCI) done 10 years ago, presented to the clinic with lower urinary tract symptoms and low back pain, with numbness, no neurological deficit, no hematuria, incontinence, retention, or flank pain. The patient was vitally stable, and on examination, prostate enlargement with a right-sided nodule was noted. Computed tomography (CT) chest abdomen pelvis (CAP) with contrast was done, which showed a prominent size of prostrate (5.2x5x3.6 cm) with small bilateral common iliac lymph nodes. There was no evidence of metastasis. In addition, a large (4.2 x 3.6 x 5.9 cm) (anteroposterior - AP, transverse, craniocaudal - CC), unruptured saccular aneurysm of the left common iliac artery located proximal to its bifurcation with partial thrombosis was incidentally discovered (Figure 1).

**Figure 2:**
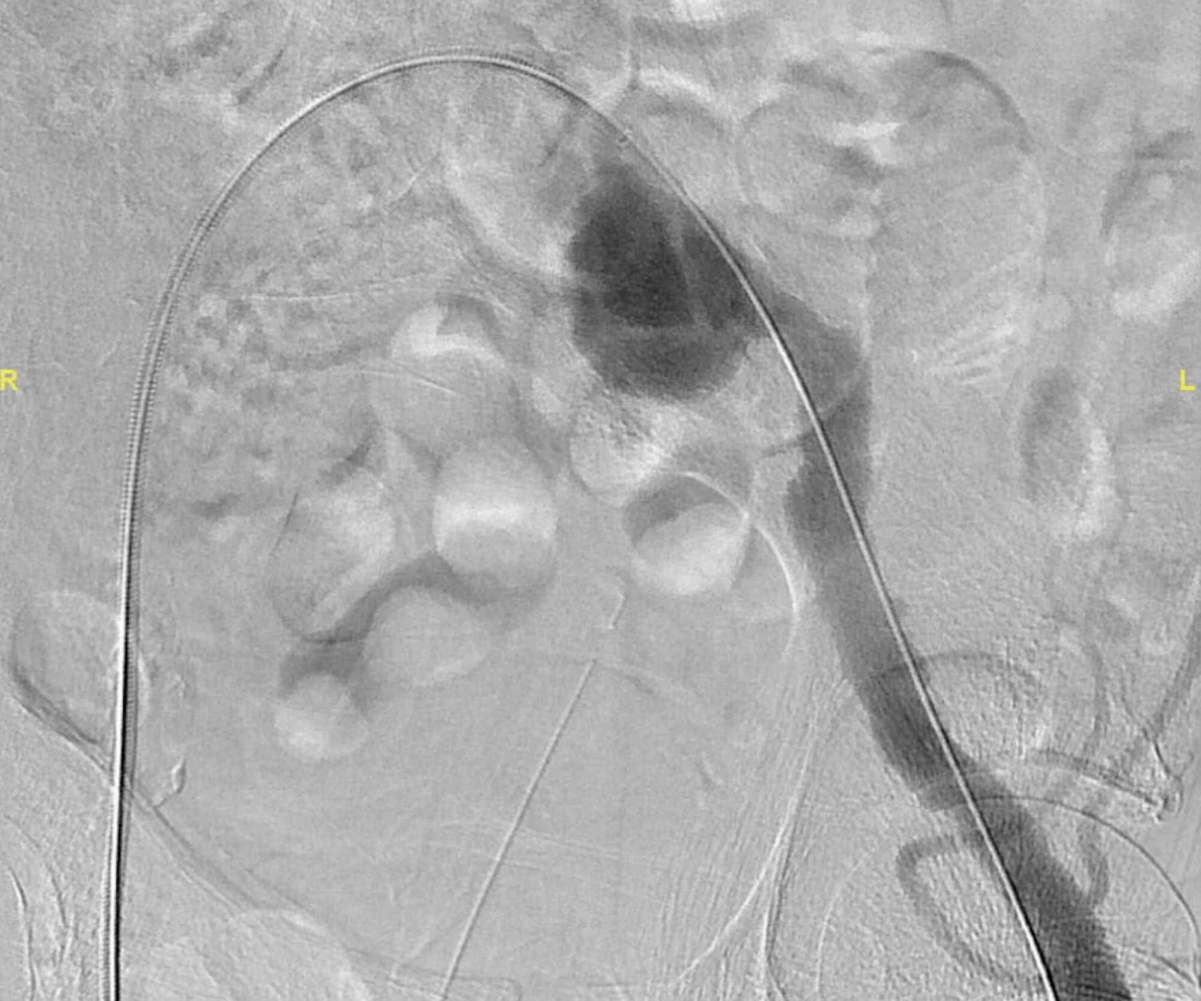
Angiogram showing a saccular aneurysm with complete internal iliac artery occlusion

Moreover, extensive atherosclerotic changes were noted in the abdominal aorta and its main branches. Left iliac artery aneurysm stent angioplasty was done under sterile conditions with ultrasound and fluoroscopy guidance. The right common femoral artery was accessed and a 6 French sheath was placed. Left common iliac artery angiogram was done, which revealed aneurysmal saccular dilatation with complete internal iliac artery occlusion (Figure 2).

**Figure 3:**
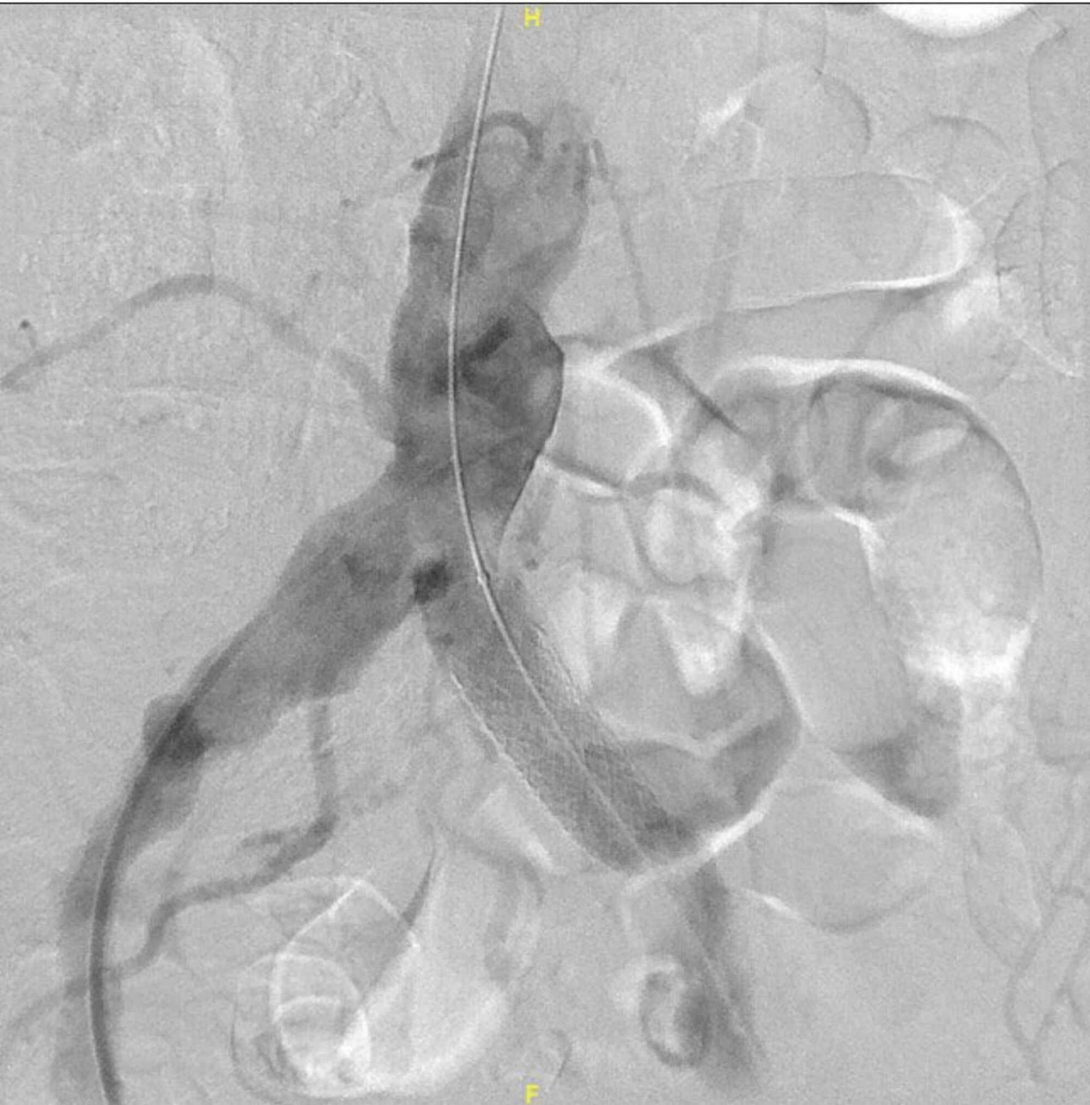
Post-angioplasty angiogram showed complete aneurysmal covering with satisfactory stent position

The left common femoral artery was accessed, and an 11 French sheath was placed successfully. A graft stent balloon mounted 14 mm and 16 mm was deployed with a complete aneurysmal ceiling. Post-angioplasty angiogram showed complete aneurysmal covering with satisfactory stent position (Figure 3).

Both femoral artery sheaths were removed, and hemostasis was achieved utilizing the ProGlide closure device (Abbott Laboratories, Abbott Park, Illinois). The patient tolerated the procedure well, with no acute complication encountered, and was discharged home.

## Discussion

IAA often coexist with abdominal artery aneurysms. Isolated iliac artery aneurysms are a rare entity, accounting for less than 2% of the total number of intra-abdominal aneurysms. In this case, the patient presented with an isolated left common iliac artery aneurysm despite the absence of an abdominal artery aneurysm, however, extensive atherosclerotic changes of the abdominal aorta were noted on the CT scan of this patient [1].

Consistent with the literature, this patient was an elderly male who had multiple other risk factors that could lead to degenerative iliac artery aneurysm, including smoking, and a history of atherosclerotic ischemic heart disease [2].

Isolated unruptured iliac artery aneurysms are often asymptomatic. In some cases, mild subjective symptoms may happen, and patients may complain of pressure symptoms that correlate with the anatomical location of the aneurysm. This patient complained of lower urinary tract symptoms as well as lower back pain and numbness, however, these symptoms could also be attributed to the presence of prostate enlargement as well as a prostate mass [1].

Physical examination in cases of isolated iliac artery aneurysms is often inconclusive, and imaging modalities help establish the diagnosis. Ultrasonography is the primary imaging modality used for the screening and diagnosis of iliac artery aneurysms and CT angiography provides better diagnostic value, as well as optimal operative planning. In this patient, a large unruptured saccular aneurysm of the left common iliac artery proximal to its bifurcation was incidentally found following the CT CAP that was initially ordered to investigate the presence of a prostate mass [5].

The endovascular approach is preferred in cases of concomitant abdominal aortic and iliac artery aneurysms, as well as in cases of isolated iliac artery aneurysms. In this patient, the endovascular approach under ultrasound with fluoroscopy guidance was chosen as a treatment modality and elective stent angioplasty was done. A graft stent balloon mounted 14 mm and 16 mm was utilized for a complete aneurysmal ceiling. Post-angioplasty angiogram showed complete aneurysmal covering and satisfactory stent position with no complications observed [5].

## Conclusions

We conclude that the early detection of an isolated iliac artery aneurysm is crucial but considered quite challenging. Moreover, surgical management can be difficult owing to its deep location. It is considered more challenging if the aneurysm is detected after rupture due to the anatomical features of the condition. Furthermore, if hemodynamic instability occurs, an exceedingly life-threatening pathology will result, making it difficult to save the patient’s life. In patients with iliac artery aneurysm discovered incidentally, prompt management is encouraged to avoid the complicated sequelae following rupture. Improving the chances of survival is possible when endovascular repair is performed rapidly.
